# Obesity: sex and sympathetics

**DOI:** 10.1186/s13293-020-00286-8

**Published:** 2020-03-11

**Authors:** Zhigang Shi, Jennifer Wong, Virginia L. Brooks

**Affiliations:** Department of Physiology and Pharmacology, L-334, Oregon Health & Science University, 3181 SW Sam Jackson Park Rd, Portland, OR 97239 USA

**Keywords:** Sympathetic nerve activity, Hypertension, Arcuate nucleus, Neuropeptide Y, Paraventricular nucleus, Leptin, Insulin

## Abstract

Obesity increases sympathetic nerve activity (SNA) in men, but not women. Here, we review current evidence suggesting that sexually dimorphic sympathoexcitatory responses to leptin and insulin may contribute. More specifically, while insulin increases SNA similarly in lean males and females, this response is markedly amplified in obese males, but is abolished in obese females. In lean female rats, leptin increases a subset of sympathetic nerves only during the high estrogen proestrus reproductive phase; thus, in obese females, because reproductive cycling can become impaired, the sporadic nature of leptin-induced sympathoexcitaton could minimize its action, despite elevated leptin levels. In contrast, in males, obesity preserves or enhances the central sympathoexcitatory response to leptin, and current evidence favors leptin’s contribution to the well-established increases in SNA induced by obesity in men. Leptin and insulin increase SNA via receptor binding in the hypothalamic arcuate nucleus and a neuropathway that includes arcuate neuropeptide Y (NPY) and proopiomelanocortin (POMC) projections to the paraventricular nucleus. These metabolic hormones normally suppress sympathoinhibitory NPY neurons and activate sympathoexcitatory POMC neurons. However, obesity appears to alter the ongoing activity and responsiveness of arcuate NPY and POMC neurons in a sexually dimorphic way, such that SNA increases in males but not females. We propose hypotheses to explain these sex differences and suggest areas of future research.

## Introduction

Obesity is an epidemic with massively broad health consequences, one of which is hypertension. It is generally accepted that a major underlying mechanism is obesity-induced activation of the sympathetic nervous system [1]. However, as recently reviewed [2, 3], while regional increases in sympathetic nerve activity (SNA) are a major contributor in males, this is generally not the case in females. More specifically, in humans, muscle SNA (MSNA) correlates to indices of obesity, like BMI or neck circumference, in men; however, in women, MSNA does not relate [2, 4–7] or relates weakly [8] to such indices. Nevertheless, there are exceptions. Obesity-induced sympathoexcitation has been observed in postmenopausal women [9], black women [10], and women with visceral obesity [11, 12].

The purpose of this brief review is to highlight recent studies that have explored whether the sexual dimorphism in obesity-induced sympathoexcitation is due to sex differences in the actions of two metabolic hormones, leptin and insulin. Plasma levels increase with obesity, and both hormones increase SNA. We begin with an overview of the neuronal pathways by which leptin and insulin increase SNA, to form a basis for the discussion of mechanistic aspects of this sexual dimorphism. As we build our model, we additionally highlight areas of future research.

## Neuronal pathways by which leptin and insulin increase SNA

### Leptin

**Fig. 1:**
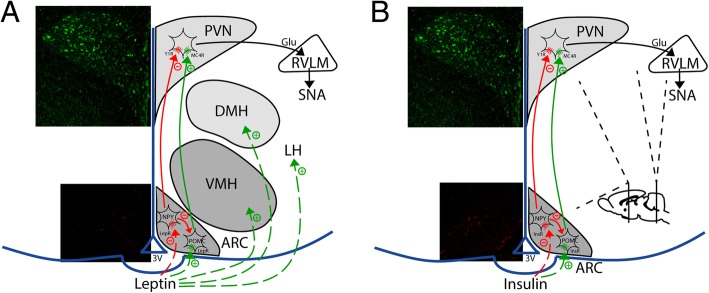
Hypothalamic sites and neuropathways by which leptin and insulin increase sympathetic nerve activity (SNA) in both males and females. **a** Leptin binds to leptin receptors (LepR) in several hypothalamic sites to increase SNA, including the arcuate nucleus (ArcN), ventromedial hypothalamus (VMH), dorsomedial hypothalamus (DMH), and lateral hypothalamus (LH). LepR binding in all these sites then triggers neuronal pathways that appear to converge in the paraventricular nucleus (PVN); ArcN neuropeptide Y (NPY) and proopiomelanocortin (POMC) neurons relay the signal from the ArcN to the PVN. Downstream, the rostral ventrolateral medulla (RVLM) is activated, which then excites preganglionic sympathetic neurons in the spinal cord. **b** Insulin acts in only one site, the ArcN, to increase SNA, via a neuropathway that includes ArcN NPY and POMC projections to the PVN, which then activate PVN glutamatergic neurons that project to the RVLM. Green (top) represents neurons that project to the RVLM. Red (**a**) is LepR. Red (**b**) is InsR

Leptin is a well-established sympathoexcitatory hormone in male animals and humans [13] (Fig. 1). It increases sympathetic drive to several organs involved in cardiovascular/blood pressure (BP) regulation, including the hindlimb [lumbar SNA (LSNA)/MSNA)], adrenal gland, kidney [renal SNA (RSNA)], as well as the splanchnic organs [splanchnic SNA (SSNA)] [14–16]. Leptin also differentially enhances baroreflex control of LSNA, SSNA, RSNA, and heart rate (HR) [14]. The increases in SNA evoked by leptin (and insulin) are homeostatically relevant, in particular after a meal: (1) insulin, and with time leptin [17–19], increase after a meal; (2) the leptin- (and insulin)-induced increases in SNA counteract direct vasodilation induced by leptin (and insulin) [20–22]; and (3) the increased muscle SNA stimulates glucose uptake [23], thereby reinforcing the direct effects of these metabolic hormones.

Recently, we discovered striking sex differences in the actions of intracerebroventricular (icv) leptin infusions on SNA in rats [24]. Leptin increased SSNA and HR similarly in males versus females throughout the estrus cycle. However, leptin activated lumbar and renal sympathetic nerves only during proestrus and in estrogen-treated ovariectomized rats, but not in ovariectomized or diestrus rats. Thus, in females, leptin requires proestrus-levels of estrogen for some of its sympathoexcitatory effects, similarly to leptin’s dependence on estrogen for its anorexic effect [25].

Is there a teleological benefit for the cyclical sympathoexcitatory effects of leptin in females? First, leptin levels are considerably higher in females than males; therefore, the dependence on elevated estrogen levels may render leptin’s sympathoexcitatory effects mute throughout much of the reproductive cycle. In addition, in young women, sympathetic activation fails to induce vasoconstriction, because of enhanced vascular β-adrenergic activity [26], and we have repeatedly found that leptin (or insulin) increases SNA without increasing BP in female rodents [24, 27]. Therefore, a hypertensive action of leptin-induced sympathoexcitation would also be nullified. Second, estrogen acts centrally to inhibit SNA [28]. Indeed, young women tend to have lower resting MSNA than men [26], due to higher estrogen levels [2]. In rats during proestrus, baseline MAP [29] and SNA (as assessed indirectly via plasma catecholamines) appear to be reduced [30], suggesting that the central sympathoinhibitory and direct vascular effects of estrogen [31, 32] are dominant. Therefore, proestrus-induced increases in leptin [33], coupled with its enhancement by estrogen, could counteract or minimize estrogen-induced vasodilation, to help maintain MAP.

The differential effects of leptin on various sympathetic nerves in males versus females suggest that diverse neuronal pathways or cellular mechanisms are involved. While leptin enhances baroreflex control of LSNA, SSNA, RSNA, and HR in both sexes, the subtly different effects between the nerves are the same in males [14] and proestrus females [24]. This result indirectly suggests that the differential effects of leptin between the sexes are not due to different sites or pathways of action, but rather a positive interaction between leptin and estrogen at the cellular level, as previous reported [34]. In support, we observed a similar dependence on estrogen for leptin’s local sympathoexcitatory effects in the paraventricular nucleus (PVN; Shi and Brooks, unpublished data). Clearly, more study is essential for a full understanding of the sex differences in the effects of leptin on SNA and how this is modified with obesity.

Our knowledge of the brain sites at which leptin increases SNA has been derived almost exclusively from studies using male rodents. Infusion of leptin into the lateral ventricle, but not the fourth ventricle, increases LSNA, suggesting that forebrain sites dominate [14]. Indeed, site-specific nanoinjections of leptin increases SNA and/or BP via multiple forebrain areas, including the arcuate nucleus (ArcN), ventromedial nucleus (VMH), dorsomedial hypothalamus (DMH), paraventricular nucleus (PVN), lateral hypothalamus (LH), and subfornical organ (SFO) (for review, see [15]) (Fig. 1). Nevertheless, leptin can, with high doses, also increase RSNA when injected into the brainstem nucleus tractus solitatius (NTS) [35]. Deletion of the leptin receptor in the ArcN eliminated intravenous leptin-induced sympathetic activation [36], suggesting that the ArcN is a major site of action.

### Insulin

Insulin is also a well-established sympathoexcitatory hormone. Insulin acts centrally to increase SNA to several vascular beds including the hindlimb, kidney, and adrenal gland, with the most profound and rapid effect on LSNA [37, 38]. Like leptin, baroreflex function is enhanced in parallel [27, 39]. Insulin also increases MSNA in humans [40, 41], as well as its baroreflex regulation [42], similarly to consuming a mixed meal. Unlike leptin, however, icv insulin infusion produces similar increases in LSNA in male rats and female rats throughout the estrous cycle [24]. Moreover, the increase in SNA induced by insulin is not diminished by ovariectomy (Shi and Brooks, unpublished results), suggesting that, unlike leptin, the response is not dependent on nor influenced by gonadal steroids. While leptin acts in several hypothalamic sites to increase SNA, and the insulin receptor is distributed throughout the brain; insulin increases SNA by binding to receptors in only one site, the ArcN, in both males and females [27, 43] (Fig. 1).

### Leptin and insulin neuronal circuitry: the ArcN-to-PVN pathway

While leptin binds to receptors throughout the hypothalamus to increase SNA, it is notable that the sympathoexcitatory response to icv leptin can be completely reversed by pharmacological blockade of the PVN [44]. These data suggest that the neuropathways emanating from each hypothalamic site converge in the PVN. Similarly, the sympathoexcitation evoked by icv or iv insulin can be completely reversed by blockade of the PVN [27, 38]. Therefore, both the PVN and the ArcN are critical nodes mediating the sympathetic effects of leptin and insulin, in both males and females.

The ArcN-PVN neural pathway is composed mainly of two components: excitatory proopiomelanocortin (POMC) neurons and sympathoinhibitory neuropeptide Y (NPY) neurons. POMC neurons release α-melanocyte stimulating hormone (α-MSH), which binds to PVN melanocortin type 4 receptors (MC4R). POMC neurons have proven to be a key element of leptin-induced sympathetic activation and hypertension development [45, 46]. Moreover, blockade of PVN MC3/4R with SHU9119 decreases LSNA in leptin-treated male rats, as well as leptin-treated proestrus rats [24, 44]. In parallel, MC4R in the PVN mediates insulin’s sympathoexcitatory actions in both males [38] and females [47]. Thus, PVN MC4R contributes to the sympathetic effects of leptin and insulin, in both males and females.

The other major component of the ArcN-PVN neural pathway is ArcN NPY neurons, which tonically inhibit sympathetic activity via release of NPY in the PVN [48]. In female rats, nanoinjection of NPY dose-dependently decreased LSNA, and RSNA, whereas PVN injection of NPY Y1 and Y5 receptor antagonists increased LSNA [48]. While NPY inputs into the PVN originate in the ArcN and brainstem, the use of designer receptors exclusively activated by designer drugs (DREADDs) to selectively activate or inhibit ArcN NPY neurons expressing agouti-related peptide (AgRP) provided evidence that this tonic NPY inhibition originates largely in the ArcN [49]. Select activation of ArcN AgRP/NPY neurons decreased SSNA, arterial pressure, and heart rate, whereas select inhibition of ArcN AgRP/NPY neurons increased SSNA, arterial pressure, and heart rate. This inhibition relied on release of NPY in the PVN and DMH. On the other hand, release of the inhibitory MC4R reverse agonist, AgRP, from ArcN neurons does not appear to be involved, since nanoinjection of AgRP in the PVN decreased body temperature, but failed to decrease SSNA, AP, or HR [49]. This result is consistent with observations in Agouti obese mice, in which the agouti peptide is ectopically expressed, but NPY expression in ArcN neurons is normal [50]: (1) Agouti obese mice exhibit elevated (rather than decreased) BP in both males and females [51, 52]; (2) in agouti obese mice, leptin failed to decrease food intake or body weight, but increased RSNA as in lean mice [53]; (3) the LSNA response to insulin was intact in the agouti obese mice [54]. In summary, AgRP released from ArcN neurons does not appear to decrease SNA and BP, like NPY.

Inhibitory NPY and excitatory a-MSH neurons converge onto the same PVN presympathetic neurons, which express both Y1R and MC4R [55–57]. We confirmed the convergence functionally: (1) all PVN presympathetic neurons (project to the rostral ventrolateral medulla) that are inhibited by NPY are also excited by α-MSH [48]; (2) the excitatory effects of PVN NPY Y1R blockade are prevented by blocking MC3/4R, in both male and female rats [48]; and (3) PVN administration of a low dose of the MC4R agonist MTII elicits sympathoexcitation only after prior blockade of NPY Y1R [44]. The latter result deserves emphasis, as it suggests that NPY acts as a “gatekeeper” for the excitatory effects of α-MSH and that the sympathetic effects of leptin and insulin require concurrent inhibition of NPY neurons with activation of POMC neurons. Indeed, leptin [44] and insulin [47] increase LSNA in part by decreasing tonic NPY inhibition in the PVN, in both males and females.

Insulin and leptin may also stimulate PVN presympathetic neurons via glutamatergic activation of ionotropic glutamate receptors (iGluR). In males, blockade of PVN iGluR partially reverses the sympathoexcitatory effects of both leptin [44] and insulin [58]. Moreover, glutamate can interact with α-MSH to increase SNA and BP [44, 59]. However, the source(s) of glutamate and the cellular mechanisms of this interaction in the PVN deserves addition investigation.

In summary, acute leptin and insulin administration increases SNA in both males and females. Due to differences in sympathetic vasoconstrictor responsiveness, central leptin and insulin increase blood pressure in males, but not females [24]. Although elevated estrogen in females can amplify leptin’s, but not insulin’s, activation of some sympathetic nerves, the same ArcN-PVN neural pathway mediates the sympathetic effects of leptin and insulin, in males and females: both decrease NPY release in the PVN [44, 47], increase α-MSH release [38, 44], and elevate glutamate drive [44, 58]. Therefore, a HFD and obesity may cause sympathetic overactivity in a sex-specific manner through its modifications of POMC neurons, NPY neurons, and PVN glutamatergic neurotransmission.

## Sex differences in effects of insulin and leptin on SNA with obesity

### Leptin

**Fig. 2:**
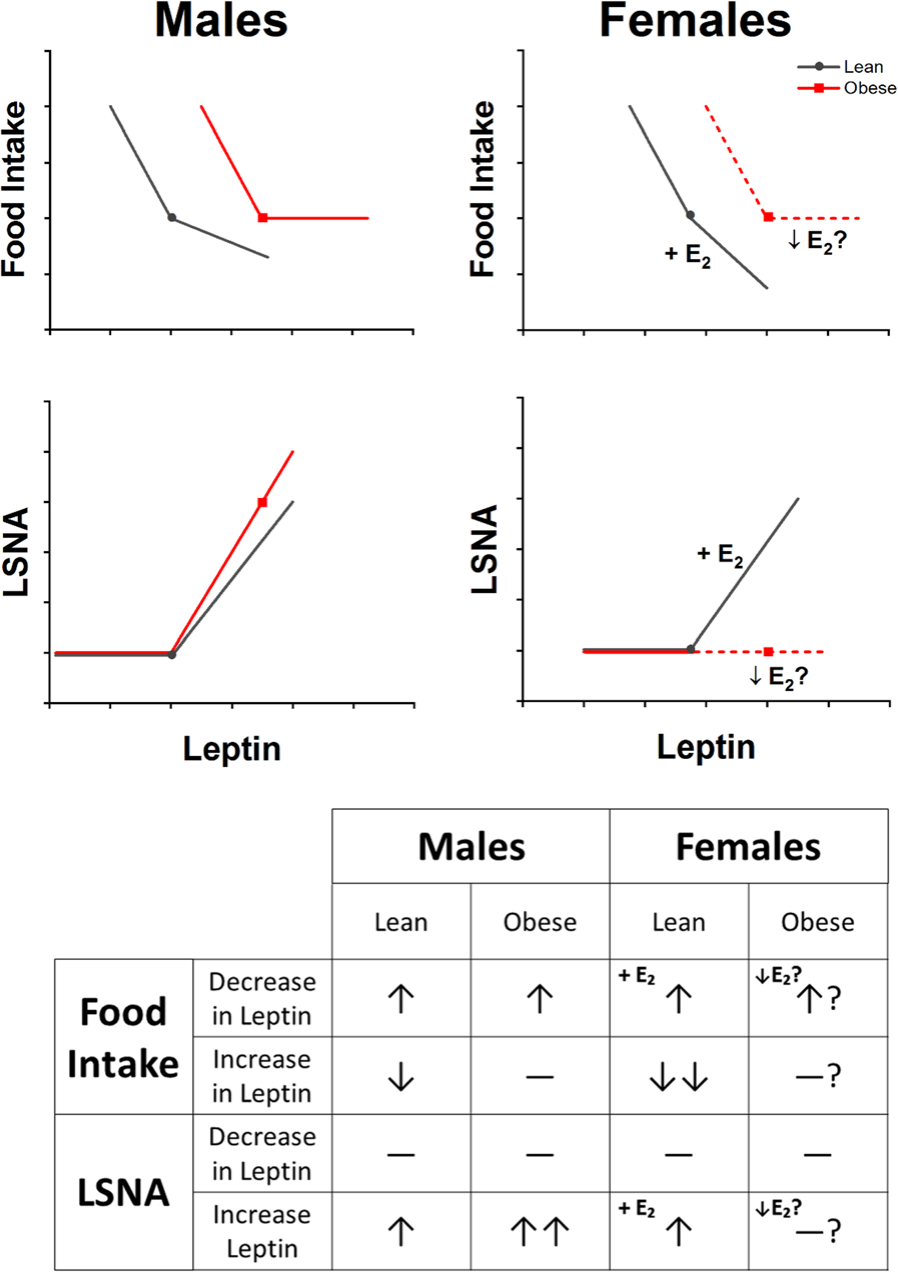
Relationships between leptin and food intake (inhibitory) and sympathetic nerve activity (SNA; excitatory) in males (left) and females (right), and how these relationships are altered by obesity. Top panel: food intake. In both males and females, a loss or decrease of leptin or leptin receptors increases food intake, producing obesity. Increases in leptin can inhibit food intake, but, in general, high doses are required. In females, leptin’s anorexic effect is enhanced by estradiol (E2). In obese males and females (red lines), the relationship between leptin and food intake is reset to a higher leptin level. In males, increases in leptin no longer inhibit food intake (“leptin resistance”), but blockade of endogenous leptin receptors increases food intake (i.e. endogenous leptin continues to suppress appetite). In females, leptin resistance takes longer to develop, presumably due to suppression of reproductive cycling and decreases in E2. However, whether endogenous leptin continues to suppress food intake in females has not been investigated. Dashed lines indicate hypothetical relationships that require further experimentation. Middle panel: lumbar SNA (LSNA): Leptin increases LSNA in both lean males and females; however, in females, this effect requires proestrus levels of E2. It is unlikely that basal levels of endogenous leptin support (L)SNA in either males or females. In obese males, leptin’s sympathoexcitatory effect is preserved or enhanced and likely contributes to the increased basal SNA observed with obesity in male humans and experimental animals. In contrast, in obese females, suppression of reproductive cycling and estrogen levels likely in parallel suppresses leptin’s sympathoexcitatory effect. This sex difference (enhanced leptin SNA increases in obese males, but reduced leptin effects in obese females), may contribute to the observation that obesity increases SNA in men, but not women. Bottom panel: Summary of known and hypothetical changes in food intake and SNA with increases and decreases in leptin levels or actions in both lean and obese males and females

One of the first recognized actions of leptin was its ability to inhibit food intake; indeed, loss of leptin or its receptor in both sexes engenders profound obesity. Conversely, increases in leptin inhibit food intake in both males and females (Fig. 2), although generally nonphysiological leptin levels are required in acute experiments. Females are more sensitive due to the facilitatory actions of estrogen [25, 60, 61] (Fig. 2). The relationship between leptin and food intake changes with obesity. In obese rodents, food intake is close to normal despite elevated leptin levels, indicating a resetting of the leptin-food intake relationship to a higher leptin level (Fig. 2). In obese males, further increments in leptin fail to inhibit food intake, i.e., leptin resistance [62], but blockade of endogenous leptin stimulates food intake as in lean animals [63] (Fig. 2). In obese female rodents, leptin resistance develops after a considerably longer time on a HFD [64], perhaps due to decreases in estrogen, which also impair reproductive cycling [65]. However, whether endogenous leptin continues to suppress food intake in obese females, as in obese males, has not been investigated.

Much less is known about whether or how obesity impacts the relationship between leptin and (L)SNA (Fig. 2). As described above, in lean males, exogenous leptin increases the activity of sympathetic nerves innervating several organs, although in acute experiments large doses are required. It is unlikely that endogenous leptin contributes to basal SNA in lean males, since icv injection of a leptin receptor antagonist does not alter RSNA in rabbits on a normal fat diet [66], and since acute blockade of the PVN or ArcN does not lower SNA. Obesity increases leptin and SNA, and as reviewed previously [45, 46], some data suggest the two are functionally related in males. First, leptin and MSNA correlate in humans [67]. Second, chronic infusion of leptin produces hypertension [46]. Third, unlike with food intake, exogenous leptin elicits a normal or enhanced SNA response in obese males, so-called selective leptin resistance [68]. Fourth, blockade of central leptin receptors in obese males reduces SNA and/or arterial pressure in some [36, 66, 69], but not all [70], studies. As described above, in lean females, increments in leptin increase LSNA only in the presence of proestrus levels of estrogen [24], and blockade of PVN MC3/4R does not decrease SNA even in proestrus rats [24]; thus, it is unlikely that leptin contributes to basal SNA in lean females as in males. In obese but normally cycling females, the variable effect of leptin on SNA during the reproductive cycle may contribute to the poor correlation of SNA to indices of obesity. Moreover, obesity can disrupt reproductive cycling in women [71] and rats [72, 73], with variable effects on estrogen levels, which may also explain in part this lack of correlation in females, unlike males. Finally, even if the obese state increases estrogen as it increases leptin, leptin may still not drive elevated SNA, because obesity in females engenders resistance to its sympathoexcitatory effects (unlike males), as it does to insulin (see below). In support, we have found that leptin fails to increase LSNA in DIO female rats in proestrus (Shi and Brooks, preliminary observations), and pregnancy, another state of added adipose, eliminates the sympathoexcitatory effects of both leptin and insulin [74]. In obese females, the development of hypertension, when it occurs, may be mediated by other factors [75].

Leptin increases SNA via actions in several brains sites, and a few studies have begun to identify which of these is engaged in obese males. More specifically, it has been shown that nonspecific blockade of the ArcN and PVN [76, 77] both decrease SNA in DIO male rats. While these results may reflect the actions of several sympathoexcitatory factors, other reports indicate that, in obese animals, select blockade of leptin receptors in the ArcN [36], VMH [78], or DMH [69] decreases SNA and/or MAP. Nevertheless, two studies were unable to confirm a role for the DMH in obesity-induced sympathoexcitation [77, 78].

In summary, considerable evidence implicates a role for leptin in obesity-induced sympathoexcitation in males. Nevertheless, other findings dispute its primacy: (1) leptin does not always correlate with MSNA in humans [7]; (2) Zucker rats, in which obesity is secondary to mutation of the LepR, still exhibit increased SNA [79], and chronic central blockade of MC3/4R decreases BP in male Zucker rats [80]; and (3) leptin is produced in the subcutaneous fat depot, but subcutaneous adiposity does not elevate MSNA [81]. Thus, other factors must also be involved in obesity-induced sympathoexcitation in males. In females, a role for leptin-induced sympathoexcitation in DIO hypertension is unlikely, but is currently unknown.

### Insulin

Obesity causes insulin resistance, albeit less in females [2], which can increase insulin and therefore SNA. Nevertheless, infusions of insulin in obese humans do not elevate MSNA [41, 82]. One interpretation of this result is that the brain, like muscle, becomes resistant to the sympathoexcitatory effects of insulin. However, as discussed in detail previously [2, 41, 83], the lack of response may be instead due to increases in plasma insulin above the capacity of the insulin transporter that mediates its movement from the plasma compartment into the brain. As a result, further increments have no effects.

**Fig. 3:**
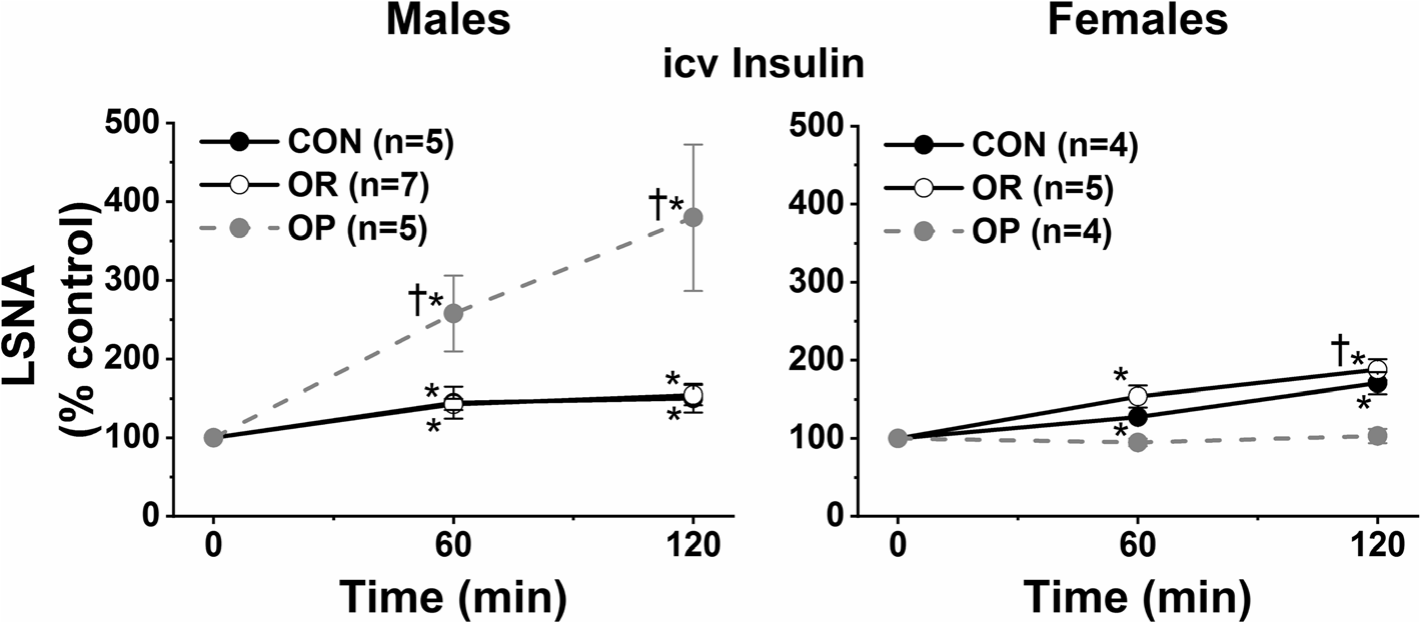
Obesity alters the sympathoexcitatory response to insulin in a sexually dimorphic way. Intracerebroventricular (icv) insulin increases lumbar sympathetic nerve activity (LSNA) similarly in lean [control diet (CON) or resistant to obesity development when consuming a high fat diet (OR)] males and females throughout the reproductive cycle. However, while obesity (obesity prone; OP) markedly amplifies the sympathoexcitatory response to icv insulin in males, in females, insulin’s action is abolished. Data adapted from [84]

To test this idea, we recently infused insulin icv in obese and lean male and female rats [84], in order to bypass the blood-brain barrier (BBB) insulin transporter (Fig. 3). We studied Sprague-Dawley rats with DIO, in which rats eating a HFD diverge into obesity-prone (OP) and obesity-resistant [OR: maintain body weight and adiposity as in control rats eating a low-fat diet (LFD)] cohorts. Importantly, the rats were studied early in obesity development (4–6 weeks on a moderate 32% HFD), before hypertension emerged. OP males were insulin resistant, but OP females exhibited only subtle signs of insulin resistance, as in humans. As previously [24], insulin increased LSNA similarly in lean (OR and control) males and females (Fig. 3). The fact that OR males and females responded like their LFD counterparts suggests that the HFD alone is not sufficient to trigger the obese phenotype. In OP males, however, the sympathoexcitatory response to insulin was amplified nearly tenfold (Fig. 3), much like the enhanced response to leptin [85]. In parallel, in the conscious state, icv insulin increased MAP only in OP male rats [77]. In sharp contrast, in OP females, icv insulin now failed to increase LSNA. These data therefore suggest another potential mechanism for why obesity increases SNA in males, but not females. In addition, the results highlight insulin as a potential factor that can drive central sympathoexcitatory pathways in obese males, despite plasma insulin levels near the maximal BBB transport activity: the brain is sensitized. Given the increasing use of intranasal insulin to treat diseases such as Alzheimer’s in humans [86], it will be important to test if this sex difference documented in rats is also present in humans (i.e., does intranasal elicit greater SNA increases in obese men, but smaller responses in obese premenopausal women?).

Additional indirect evidence also implicates insulin in obesity-induced sympathoexcitation in males: (1) icv injection of an InsR antagonist decreases BP in obese rabbits [66]; (2) in obese humans, MSNA correlates with insulin resistance and elevated insulin levels in men, but not women [7]; and (3) weight loss decreases MSNA and NE spillover in humans and this correlates with the fall in plasma insulin, regardless of glycemic status [87]. Our recent studies provide direct evidence; nonspecific blockade of the ArcN (the sole site at which insulin increases SNA [27, 43]) or acute select blockade of ArcN InsR decreased LSNA and BP in male OP, but not OR/control, rats [77]. Thus, insulin also contributes to obesity-induced activation of central sympathoexcitatory pathways in males. Importantly, the rats were studied only after 4–6 weeks of a HFD, before developing hypertension. Therefore, the data reinforce the concept that increases in SNA evolve early in subjects on a HFD [88, 89], before blood pressure increases. These preclinical results mirror the human condition; obesity increases SNA, but not always BP, because, in obese individuals, the vasculature resists norepinephrine-induced vasoconstriction [90–92].

In summary, current evidence suggests that obesity increases SNA in males, but not females, in part because of differential responsiveness to insulin. Nevertheless, a definitive experiment, which determines if chronic central InsR blockade decreases BP and SNA in obese hypertensive males, but not females, is lacking.

#### Mechanisms for differential responses to leptin and insulin in obese males and females

**Fig. 4:**
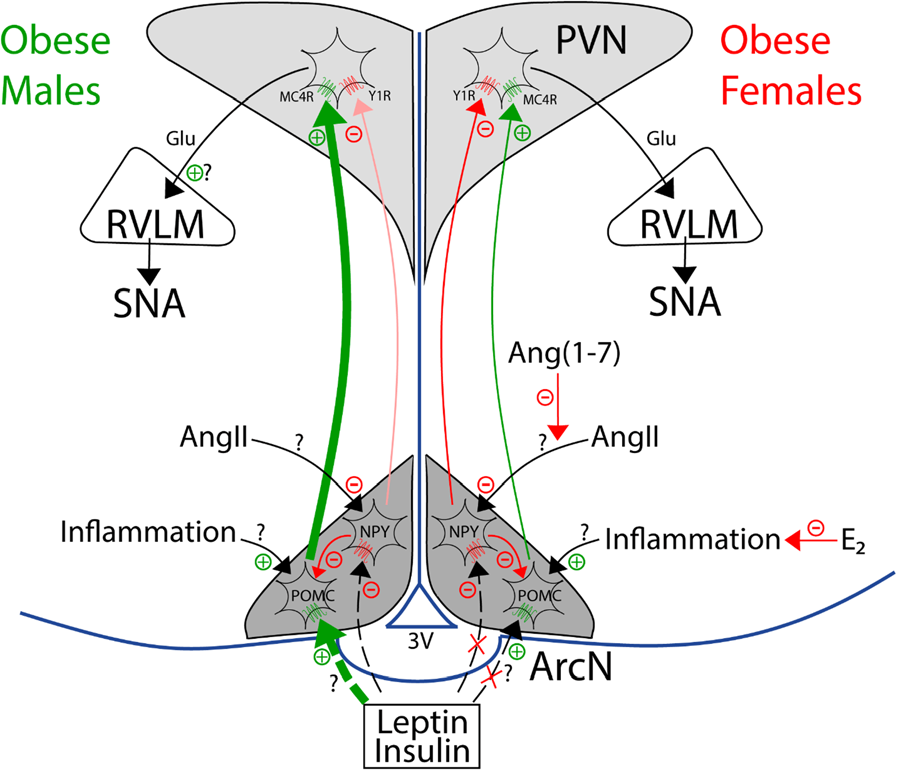
Proposed mechanisms to explain why obesity enhances the sympathoexcitatory response to insulin (and perhaps leptin) in males, but abolishes these responses in females. Left, males. In obese males, increased basal sympathetic nerve activity (SNA) is driven in part by suppressed tonic Neuropeptide Y (NPY) sympathoinhibition, and increased α-melanocyte stimulating hormone (α-MSH) excitation, of paraventricular (PVN) presympathetic neurons. Because the increase in SNA in response to PVN α-MSH agonists, like MTII, are the same in otherwise untreated obese male rats and in lean rats after blockade of PVN NPY Y1 receptors, the increased basal tone may be explained simply by the loss of tonic NPY inhibition of PVN presympathetic nerves and/or increased signaling upstream in arcuate nucleus (ArcN) proopiomelanocortin (POMC) neurons. In addition, therefore, the amplified SNA response to insulin in obese males is likely due to increased signaling in insulin-receptive POMC neurons. The mechanism for the enhanced insulin responsiveness is unknown, but may involve local ArcN actions of Angiotensin II (AngII) to inhibit NPY neurons and/or the inflammatory mediators like cytokines to excite POMC neurons. Right, females. In females, basal SNA may not increase, because tonic NPY sympathoinhibition is maintained and not inhibitable by insulin. The maintenance of NPY tone may be explained by the hypertensive actions of AngII becoming neutralized by the antihypertensive actions of Ang-(1-7), as in the periphery. However, future research is needed to address many of these mechanistic links in both obese male and female rodents, and when possible, in men and women

As detailed above, both leptin and insulin increase SNA by simultaneously suppressing NPY inputs to the PVN, which tonically inhibit SNA, and activating POMC neuronal inputs, which release the sympathoexcitatory peptide α-MSH. Therefore, in males, the exaggerated responses to leptin and insulin could be secondary to decreased NPY inhibition and enhanced POMC excitation (Fig. 4).

We recently tested if NPY sympathoinhibition is suppressed in obese males [84]. In lean animals, blockade of PVN NPY Y1R increases SNA (NPY tonically inhibits SNA) [48]. However, in obese males, PVN NPY Y1R blockade had no effects; tonic NPY inhibition was lost. Yet, the sympathoinhibition elicited by PVN NPY nanoinjections was the same in lean and obese male rats, indicating that the loss of PVN inhibitory NPY influences was not due to a decrease in NPY Y1R expression or signaling. Instead, obesity eliminates tonic NPY inhibitory inputs to the PVN in males, allowing excitatory inputs, like α-MSH, to increase SNA, unfettered by inhibition.

Indirect evidence supporting a role for enhanced α-MSH actions in PVN includes the findings that acute or chronic blockade of brain MC3/4R with icv SHU9119 [93, 94], and that nonspecific blockade of the PVN [76], decrease SNA and BP male rats with DIO. More recently, we reported that acute blockade of PVN MC3/4R decreased LSNA in OP but not lean rats [95], providing direct support. Interestingly, icv [94] or PVN [95] injections of MTII or α-MSH, peptides that activate MC4R, increased SNA only in obese animals. The increase in SNA in response to MTII in OP rats (4–6 weeks of a HFD) was the same as the increase in SNA to MTII after blocking PVN NPY1R in lean rats [44]. Because tonic NPY inhibition is absent in OP rats [84], these results suggest that the emergence of PVN MTII responsiveness in this early stage is due solely to the loss of tonic NPY inhibition, rather than a change in MC3/4 receptor content or responsiveness in the PVN. Moreover, it appears, at least in this early stage, that obesity does not upregulate downstream mechanisms, as in the brainstem [96]. Therefore, coupled with the finding that obesity does not increase InsR expression in the ArcN [84, 95, 97], the markedly amplified increases in SNA evoked by insulin must be secondary to increases in insulin-induced cellular signaling in the ArcN.

The actions of PVN NPY and α-MSH in OP female rats were completely different from obese males [84]. In females, tonic inhibition of SNA via PVN NPY Y1R was the same in obese and lean rats. More importantly, while icv insulin suppressed this inhibition in lean females, it did not in obese females; ArcN NPY neurons were resistant to the inhibitory effects of insulin [84]. Interestingly, we also found that, after NPY Y1R blockade, insulin did not increase SNA nearly as much in obese females as lean females. Because the sympathoexcitatory response to insulin depends on α-MSH in the PVN [38, 47], this result indirectly suggests that insulin’s activation of ArcN POMC neurons is also weakened with obesity in females. However, future experiments are required to test this hypothesis.

#### Mechanisms for the differential impact of obesity in the ArcN in males versus females

Collectively, these data highlight the role of the ArcN, specifically POMC and NPY neurons, as a site of sexual dimorphism in obesity-induced sympathoexcitation in OP versus OR/control males and females. We will next consider possible mechanisms for these different responses. We acknowledge that the mechanisms underlying greater sympathoexcitation in obese males compared to females are complex and likely vary among the various animal models and human populations. Therefore, our focus will be on rodents consuming a HFD, with information from other models included where relevant.

##### Males

DIO males can exhibit enhanced sympathetic responses to both leptin [85] and insulin [84]. In an important study of obese rabbits, the Head lab [85] demonstrated that while icv leptin induced greater increases in RSNA, neuronal activation (as assessed via c-fos induction) was markedly reduced in many hypothalamic (and other brain) nuclei. This finding reaffirms that while obesity renders most leptin-responsive neurons insensitive, those neurons involved in autonomic control are spared or even sensitized, “selective leptin resistance.” This result also indicates that it will be challenging to identify specific cellular mechanisms of sensitization, because the autonomic population of neurons in the ArcN (and other sites) is such a small fraction of all neurons containing LepR/InsR or influenced indirectly by leptin or insulin. Therefore, what follows is our speculation on potential or hypothesized mechanisms, awaiting further experimentation.

It is well established that, in males, DIO activates the renin-angiotensin system (RAS) [98, 99] and induces inflammation [100]. Moreover, blockade of the RAS prevents or largely attenuates the pressor or sympathoexcitatory responses to both leptin [101] and insulin [102–105], suggesting a dependence or potential for interaction. In a recent series of experiments, the Johnson/Felder labs have highlighted the importance of an obesity-induced synergism between leptin, the RAS, and inflammation in the brain sensitization to hypertensive stimuli (and other pathological stresses) (see [106] for a review). Briefly, they showed that 3 weeks of a 60% HFD upregulated brain leptin receptors, RAS components, and indices of inflammation, without producing hypertension [107]. Subsequently, after a 1 week return to a LFD, this HFD priming significantly enhanced the hypertensive response to a systemic, normally nonpressor, AngII infusion. Interestingly, central LepR blockade prevented HFD sensitization and icv leptin or TNF-α infusion mimicked HFD sensitization [107]. Therefore, they concluded that in DIO rats, central interactions between leptin, the RAS (in particular AngII and AT1R), and inflammation set the stage for the hypertension induced by systemically administered AngII. A key remaining question is whether the AngII-induced hypertension in HFD-primed rats is mediated by the amplified central actions of AngII to increase SNA or to an interaction between the systemic pressor actions of AngII (which are increased in obese males) and central mechanisms. Remarkably, only one study has tested if central AT1R blockade or elimination prevents or minimizes hypertension in DIO males [108]. In mice on a HFD (4 weeks), deletion of AT1aR in the PVN lowered systolic pressure, but only in the dark phase and in association with a substantial decrease in motor activity. Clearly, more work is needed to establish a role for AT1aR/AngII in obesity-induced sympathoexcitation in males.

In OP males, tonic NPY inhibition of SNA is eliminated, whereas insulin-induced stimulation of POMC neurons appears to be enhanced [84]. Can increased AngII (via AT1R) or inflammation underlie the reduced NPY tone or increased sensitivity of POMC neurons to insulin? In support of a potential role for AT1R, the ArcN expresses AT1R in both males and females [109, 110]. Moreover, ArcN nanoinjections of AngII increase SNA and BP [111, 112]. Both a HFD [113] and AngII [114] suppress ArcN NPY expression. Recently, ArcN AT1R were identified largely in NPY/AgRP neurons in mice [115]; in the rat, AT1R were also found in NPY neurons, albeit at lower levels, and again rarely found in POMC neurons (Pelletier and Brooks, unpublished information). Like insulin and leptin, ArcN AngII increases SNA via inhibition of NPY neurons and activation of POMC neurons and release of α-MSH in the PVN [116]. The limited expression of AT1R in POMC neurons suggests that this excitation involves a local interneuron, but the exact mechanisms require further study. Claflin et al [115] also tested the role of AT1R in the suppression of NPY by a HFD. They reported that HFD feeding decreased hypothalamic AgRP mRNA expression in control mice, but not in mice in which the AT1R was deleted in LepR-containing neurons (the AT1R and LepR are co-expressed in the ArcN) [115], supporting a link between AT1R and NPY suppression with obesity. Thus, it is possible that an increased action of AngII at AT1R could suppress tonic NPY sympathoinhibitory tone in obese males, but this hypothesis awaits further study.

How does obesity amplify the actions of insulin (and leptin) on POMC neurons in males? Inflammation is another sensitizing factor, which is well described in the ArcN of males with DIO [117] (for reviews, see [100, 118, 119]). Inflammation is evoked by both a direct action of saturated free fatty acids (SFA) on microglial cells in the hypothalamus and by infiltration of immune cells from the periphery [117]. Cellular mediators of inflammation include IKK-β and NF-κB [120]. Knockdown of IKK-β ameliorates hypertension in male mice with DIO [121]. Interestingly, the cytokine TNF-α activates IKK-β in POMC neurons far more than AgRP/NPY neurons [121]. Moreover, selective knockdown of IKK-β in POMC neurons prevented DIO-induced hypertension, whereas IKK-β knockdown in AgRP/NPY neurons was ineffective [121]. Collectively, these data support the hypothesis that in obese males, activation of the RAS and increased ArcN AT1R activity, suppresses tonic NPY sympathoinhibition, whereas local inflammation in the ArcN may sensitize POMC neurons to sympathoexcitatory factors, like insulin. Future experiments are required to test these hypotheses.

##### Females

As described above, unlike obese males that exhibit an enhanced SNA response to insulin, in obese females, insulin-induced increases in SNA were eliminated, in part because activation of POMC neurons appeared muted [84]. Can this be due to less inflammation in obese females? Considerable work supports this hypothesis. First, in premenopausal women, estrogen acts via ERα to favor the distribution of adipose preferentially to the subcutaneous compartment [122]. In contrast, during menopause (or following ovariectomy), more fat is deposited in the visceral compartment. Visceral fat is more inflammatory [2]. Therefore, females by virtue of their adipose distribution tend to exhibit less inflammation, a potential sensitizer of POMC neurons. Second, estrogen is anti-inflammatory at several levels, including immune cells, adipose tissue, and the brain [123, 124]. Third, a HFD is inflammatory in part via the activation of brain microglia by saturated fatty acids (SFA) [125]. Females on a HFD differentially metabolize fat, by producing less SFA, instead producing more favorable unsaturated fatty acids [124]. Brain estrogen receptor alpha is anti-inflammatory, but is reduced in males by HFD-derived SFA [124]. Indeed, a HFD elicits less microglial activation in females [120]. Thus, a hypothesis to be tested is that in OP females, POMC neurons are not sensitized to insulin, because of less inflammation. Indirect support incudes the findings that obese postmenopausal women or women with visceral obesity exhibit elevated MSNA [9, 11, 12]. Alternatively, because NPY neurons can directly inhibit POMC neurons within the ArcN (Fig. 4), maintained NPY tone could render POMC neurons less responsive to other stimuli.

Another sex difference was that tonic NPY sympathoinhibition was maintained in OP females [84]. Can this be due in part to less inhibition by AngII in the ArcN? In support, as reviewed previously [2, 28, 126, 127], (1) in males, obesity increased components of the hypertensive arm of the RAS, AngII, and AT1R, but decreased components of the antihypertensive arm, Ang-(1-7) and ACE2. In females, obesity had the opposite effects (increased Ang-(1-7) and ACE2; no increase in AngII) [99]. Moreover, ArcN Ang-(1-7) can decrease SNA and BP in females (Shi and Brooks, unpublished data); (2) in brain, estrogen upregulates the antihypertensive pathway and inhibits the hypertensive RAS pathway [28]; and (3) ArcN AngII receptor binding varies with the estrus cycle: highest during estrus and nearly undetectable during proestrus [128]; treatment of ovariectomized rats with sequential estrogen/progesterone injections (to mimic the estrus phase) markedly increased AT1R expression [110]. In parallel, we have found that ArcN AngII nanoinjections produce the greatest increases in SNA during estrus; ArcN AngII is ineffective during proestrus (Shi and Brooks, unpublished findings). Interestingly, while obesity has been associated with reproductive cycle disruption [72, 73] and increases or decreases in estrogen [71], a common feature is a decrease in progesterone [129]. Thus, a hypothesis to be tested is that obesity-induced falls in progesterone, especially if associated with increases in estrogen, decreases ArcN AT1R expression, and its ability to suppress NPY sympathoinhibition. Future work is required to determine if reduced AT1R activation or the laudatory effects of systemic Ang-(1-7) and ACE2 in obese females are duplicated in the ArcN, specifically if by counteracting the inhibition of NPY neurons by AngII.

Finally, what are the mechanisms by which ArcN NPY and POMC neurons become resistant to insulin in OP females? This does not appear to be due to a decrease in the expression of InsR in NPY neurons [84]; therefore, obesity in females somehow attenuates insulin signaling specifically in presympathetic ArcN neurons. Since functional AT1aR are required for insulin and leptin sympathoexcitatory and hypertensive effects [101–105], a reduction in ArcN AT1aR expression or signaling could contribute. Again, future experiments are required.

## Conclusions

In lean male and female rodents, leptin and insulin both increase SNA via receptor binding in the ArcN and a neuropathway that includes alterations of ArcN inputs to the PVN (Fig. 1): suppression of tonic NPY sympathoinhibition and stimulation of POMC sympathoexcitation (via release of α-MSH). However, while the ArcN is the sole site at which insulin elicits increases in SNA, leptin acts in several hypothalamic sites, all of which appear to converge in the PVN. There are subtle differences that limit the actions of leptin/insulin to increase SNA and/or BP in lean females: (1) leptin only increases LSNA and RSNA during proestrus, through the synergistic actions of elevated levels of estrogen, and (2) the increases in SNA evoked by central leptin and insulin cause vasoconstriction and increase BP in males, but not females.

Obesity increases SNA in men, but only rarely in women. One potential mechanism may involve sexually dimorphic changes in the central actions of insulin and leptin with obesity (Fig. 4). The sympathoexcitatory response to insulin is markedly amplified in obese males, but abolished in obese females. Obesity also preserves or enhances the central sympathoexcitatory response to leptin in males; while not yet investigated, because reproductive cycling becomes impaired, obesity may also further limit leptin-induced sympathoexcitation in females. These changes appear to be due to sexually dimorphic alterations in NPY and POMC inputs to the PVN (Fig. 4). In obese males, tonic PVN NPY sympathoinhibition is lost, and POMC neuronal input to the PVN increases, possibly due in part to increased insulin-induced ArcN POMC cellular signaling. In contrast, in obese females, tonic NPY sympathoinhibition is maintained and not inhibitable by insulin; POMC sensitivity to insulin also may decrease. The mechanisms for these sexually divergent changes induced by obesity are not known, but we speculate that greater inhibition of NPY neurons via the hypertensive, versus non-hypertensive, arms of the RAS, and the greater inflammatory excitation of POMC neurons, in obese males versus females may contribute. However, the mechanisms by which the effects of insulin (and possibly leptin) on ArcN NPY or POMC neurons become muted in obese females are unknown.

## Perspectives and significance

It must be acknowledged that much of our current understanding and proposed testable hypotheses are based on acute experiments in anesthetized rodents, which may not exhibit all the features of human obesity, like obstructive sleep apnea and aging. Therefore, going forward, it will be critically important to test various aspects of the model in humans and under more chronic conditions in awake animals. Some potentially impactful studies in humans could address the following questions: (1) does intranasal insulin increase MSNA more in obese men, than in obese premenopausal women? (2) Is there greater activity of the hypertensive versus antihypertensive arm of the RAS in obese men than women, which increases blood pressure and MSNA more? (3) While RAS blockade has been shown to decrease MSNA in obese men and women [130], sex differences were not examined; therefore, does inhibition of the RAS (ACEI vs ARB) decrease MSNA more in obese men than premenopausal obese women without visceral obesity? (4) Is the sympathoexcitatory effect of progesterone (or the progesterone:estrogen ratio) during the menstrual cycle [131] altered by obesity? In preclinical studies, more work is needed to determine (1) the potential sites and mechanisms by which the various brain estrogen receptors, as well as progesterone and its neurosteroid metabolite, allopregnanolone, are involved in the sympathoexcitatory effects of obesity and leptin/insulin; (2) the brain sites and mechanisms by which the RAS (both hypertensive and antihypertensive components) interacts with leptin/insulin to increase SNA and how this differs in lean and obese males versus females; (3) the brain sites and mechanisms by which inflammation can increase SNA in males versus females; and (4) whether inflammation and the RAS contribute differentially to the sex differences in the impact of obesity in the ArcN.

## Data Availability

Not applicable.
